# TRACKING OF CARDIORESPIRATORY FITNESS FROM CHILDHOOD TO EARLY ADOLESCENCE: MODERATION EFFECT OF SOMATIC MATURATION

**DOI:** 10.1590/1984-0462/;2019;37;3;00015

**Published:** 2019-05-09

**Authors:** André Oliveira Werneck, Danilo Rodrigues Silva, Ricardo Ribeiro Agostinete, Rômulo Araújo Fernandes, João Valente-dos-Santos, Manuel João Coelho-e-Silva, Enio Ricardo Vaz Ronque

**Affiliations:** a Universidade Estadual de Londrina, Londrina, PR, Brazil.; b Universidade Estadual Paulista “Júlio de Mesquita Filho”, Presidente Prudente, SP, Brazil.; c Universidade de Coimbra, Coimbra, Portugal.

**Keywords:** Puberty, Physical fitness, Sexual maturation, Motor activity, Youth, Puberdade, Aptidão física, Maturidade sexual, Atividade motora, Adolescente

## Abstract

**Objective:**

To evaluate cardiorespiratory fitness’ tracking from childhood to adolescence, as well as to test the moderation role of somatic maturation.

**Methods:**

Our sample was composed by 375 children (197 boys), with a baseline age between 7 and 10 years old. The children were followed-up over three years. Body mass and stature were measured as anthropometric indicators and were used to estimate maturity status through Moore’s method. Cardiorespiratory fitness was evaluated through 9-minute running test. Body adiposity was estimated through the subcutaneous skinfold method, with measures of triceps and subscapular skinfolds and used as a covariate. Sample was categorized into tertiles. Thereafter, the *Kappa* (k) coefficient and Lin’s concordance correlation coefficient (LCCC) tests were adopted to verify stability. Dummy variable in regression was used to test moderation effects. All analyses were conducted in Stata 14.0, adopting p<0.05.

**Results:**

Cardiorespiratory fitness presented a moderate to low tracking from childhood to adolescence (k=0.294; LCCC=0.458). Moreover, maturity status significantly moderated the association between cardiorespiratory fitness at childhood and adolescence (regardless of cohort and body adiposity) among boys (β=0.644; p=0.003) and role sample (β=0.184; p=0.020), but not girls (-0.217; p=0.413).

**Conclusions:**

Tracking of cardiorespiratory fitness from childhood to adolescence is moderate to low in both sexes. Moreover, maturity status moderated the relationship between cardiorespiratory fitness at baseline and in adolescence. A lower age at peak height velocity was associated to a greater cardiorespiratory fitness.

## RESUMO

**Objetivo:**

Avaliar o *tracking* da aptidão cardiorrespiratória da infância à adolescência, bem como testar a via de moderação pela maturação somática.

**Métodos:**

A amostra foi composta de 375 crianças (197 meninos), com idades entre 7 e 10 anos na linha de base. Os sujeitos foram acompanhados por três anos. Massa corporal e estatura foram mensuradas como indicadores antropométricos e usadas para estimar a maturação somática pelo método de Moore. Aptidão cardiorrespiratória foi avaliada pelo teste de 9 minutos de corrida. Adiposidade corporal foi estimada pelo método de dobras subcutâneas, com medidas das dobras tricipital e subescapular, além de ter sido utilizada como covariável. A amostra foi categorizada em tercis, e então o coeficiente *Kappa* (k) e o coeficiente de correlação de concordância de Lin (LCCC) foram adotados para verificar estabilidade. Foram criadas variáveis *dummy* nas regressões para estimar o efeito de moderação. Todas as análises foram conduzidas no Stata 14.0, adotando p<0,05.

**Resultados:**

A aptidão cardiorrespiratória apresentou *tracking* baixo a moderado da infância para a adolescência (k=0,294; LCCC=0,458). Além disso, o *status* de maturação moderou significativamente a associação entre aptidão cardiorrespiratória na infância e adolescência (independentemente da coorte e adiposidade) em meninos (β=0,644; p<0,003) e na amostra total (β=0,184; p<0,020), mas não em meninas (-0,217; p=0,413).

**Conclusões:**

O *tracking* da aptidão cardiorrespiratória da infância à adolescência é baixo a moderado em ambos os sexos. Ainda, o estado maturacional moderou a associação entre aptidão cardiorrespiratória na infância e adolescência. Menor idade do pico de velocidade de crescimento foi associada à maior aptidão cardiorrespiratória.

## INTRODUCTION

Cardiorespiratory fitness, defined as the overall capacity of the cardiovascular and pulmonary systems to transport and use oxygen during exercise (aerobic capacity), is an important component of physical fitness and is recognized as a protection factor for chronic diseases and all-cause mortality.^1^ ^,^ ^2^ ^,^ ^3^ Even during childhood and adolescence, higher levels of cardiorespiratory fitness are inversely associated with metabolic risk, regardless of physical activity practice.^4^ In addition, cardiorespiratory fitness mediates the relationship between obesity and metabolic risk in the transition between childhood and adolescence.^5^ However, considering that cardiorespiratory fitness is a complex result of hereditary and environmental predictors,^6^ comprehension of possible mechanisms for its maintenance from childhood to adolescence should support interventions for health promotion from early ages.

Among the modifiable factors associated with cardiorespiratory fitness, interventions with physical exercise have been the main strategy, aimed at maintenance and development. However, especially during adolescence, growth and maturation processes exert an important influence on different components of physical fitness.^7^ For example, even controlling for physical activity, tracking of physical fitness seems to be low^8^ and it is possible that there is a “trigger point” for the development of cardiorespiratory fitness, as well as other physical fitness domains, that often occurs during puberty.^9^ Still, the most consistent evidence points to a pubertal effect on cardiorespiratory fitness, especially among boys,^10^ in which increases in lean body mass, are observed following maturation of ventricular, arterial, and lung functions, improving cardiorespiratory fitness. On the other hand, although girls also present improvements in cardiovascular function during puberty, one of the main effects of the maturation process (female sexual hormones) is an increase in relative body fat mass, impacting on performance in tests that demand body displacement.^7^ Therefore, even the association between biological maturation and aerobic fitness being well stablished in cross-sectional studies,^10^ ^,^ ^11^ evidence of the association how biological maturation can influence aerobic fitness from childhood to adolescence is not clear, especially among girls.

In this sense, biological maturation could affect or even moderate the relationship between cardiorespiratory fitness in childhood and adolescence. Thus, our aim was to evaluate cardiorespiratory fitness from childhood to adolescence, as well as to test the moderation role of somatic maturation. The initial hypothesis of this study was that tracking of cardiorespiratory fitness could be influenced by maturational status in opposite senses in boys and girls.

## METHOD

This was a longitudinal mixed study with four birth years of cohorts (1992, 1993, 1994, and 1995). Aged between 7 and 10 years old at baseline, participants were followed-up over three years. The local ethics committee, according to the declaration of Helsinki, approved all procedures. More information about the methods and sampling can be found in the baseline study, which was published previously.^12^

The data were collected from a private school in the downtown of a city in Brazil, in which students have high socioeconomic conditions,^12^ to attend the sampling criteria (simple random sampling, adopting: α=95%, statistical power=80%, and error of 5%).^12^ Inclusion criteria were:

- Being enrolled in the school.
- Being the established chronological age (7-10 years old).
- Having interest in participating in the study.
- Presenting the consent term signed by the parents.

All students from the school that attained inclusion criteria were invited to participate in the study. In this sense, the initial sample was composed by 510 adolescents (267 boys). However, due to losses during the follow-up, 375 subjects (197 boys) completed the tests during three years of follow-up. Moreover, three participants with incomplete data were excluded. Thus, the final sample was 372 adolescents (196 boys and 176 girls).

Height was measured using a stadiometer and body mass using an electronic scale (Filizola^®^, Recife, Brazil). The date of birth and day of assessment were used to calculate chronological age. Triceps and subscapular skinfolds were collected using a Lange caliper (Cambridge Scientific Industries, Inc., Cambridge, Maryland, United States; range: 0-60 mm × 1 mm), according to procedures described by Harrison et al.^13^ (right side of the body), and performed by a single, trained assessor. Based on the measures of triceps and subscapular skinfolds, results were interpreted through the sum of skinfolds.

A 9-minute running or walking field test was performed on an official athletics track, according to the recommendations of Cooper et al.^14^ This is an adaptation for children and adolescents of the original 12-minute test. The subjects were oriented to walk and/or run the maximum distance possible in 9 minutes. Final distance was used as an indicator of aerobic fitness. To verify tracking, the sample was divided into terciles based on the 9 minutes of running/walking according to sex and age, using percentiles as cut-points (33.3 and 66.6:[≤Pº 33.3 tercile 1; >Pº 33.3 but ≤Pº 66.6 tercile 2; >Pº 66.6 tercile 3]).

Biological maturation was estimated through the somatic maturation method proposed by Moore et al.,^15^ using data from the final year of follow-up. This method estimates maturity-offset (in years) from height and chronological age using the Equation 1 for boys:

Maturity offset for boys (years) = -7.999994 + (0.0036124 × (age × height)) (1)

And using the Equation 2 for girls:

Maturity offset for girls (years) = -7.709133 + (0.0042232 × (age × height)) (2)

Then, a result is given in years from peak height velocity (PHV). Age of PHV was determined by subtracting the maturity offset from chronological age. Participants were classified as late, early or on time maturation through the one standard deviation method. Those who presented an age of PHV higher than 1 standard deviation were classified as *late*; between -1 and +1 as *on time*; and those with an age of PHV lower than 1 standard deviation were classified as *early*.

To characterize the sample, we used mean and standard deviation values. The Mann-Whitney test was used to compare groups, and chi-square test was performed for trend of somatic maturity categories according to sex. Tracking was analyzed through the application of two statistical procedures:

- To verify agreement (tracking) between proportions of subjects - the *Kappa* coefficient (k) was used (k<0.20 was considered low; k between 0.41 and 0.60, moderate; k between 0.61 and 0.80, high, and k>0.80, very high).^16^
- Lin’s concordance correlation coefficient (LCCC).

To verify the moderation by somatic maturation, we kept all variables continuous and used an interaction term between cardiorespiratory fitness at baseline and somatic maturation to predict cardiorespiratory fitness after the three-year follow-up. The significance level adopted was p< 0.05.

## RESULTS

The sample was composed of 372 adolescents (196 boys), aged between 7 and 10 years old at baseline. Table 1 presents characteristics of the sample according the sex. In general, boys matured later and present higher distance of 9-minute running tests in all sections of the study (p<0.001). Moreover, girls presented greater body adiposity at baseline than boys (p<0.05).

**Table 1:** Characteristics of the sample.

	Boys (196)	Girls (176)	p-value
Chronological age at baseline (years)	8.9±1.1	9.0±1.1	0.751
Age at peak height velocity (years)	13.1±0.4	11.9±0.3	<0.001
Somatic maturity status			0.615
Late maturing (n)	36	31	
On time maturing (n)	129	123	
Early maturing (n)	31	22	
9-min running baseline (m)	1334.5±172.6	1223.2±172.6	<0.001
9-min running 1 year (m)	1355.0±228.7	1220.1±200.8	<0.001
9-min running 2 years (m)	1392.2±218.1	1230.1±186.8	<0.001
9-min running 3 years (m)	1561.9±342.0	1334.9±232.5	<0.001
Sum of skinfolds* baseline (mm)	23.5±11.8	25.7±10.8	0.003
Sum of skinfolds* 3 years (mm)	33.5±16.3	33.9±13.6	0.206

*Triceps and subscapular skinfolds.

**Figure 1:**
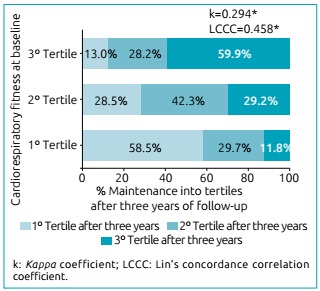
Tracking of 9-minute running distance from childhood to adolescence.

**Figure 2:**
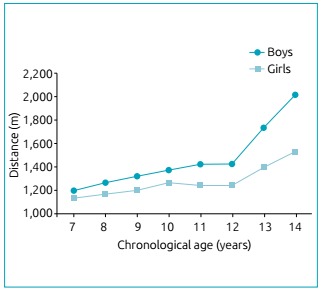
Nine-minute running distance for chronological ages according to sex.

**Figure 3:**
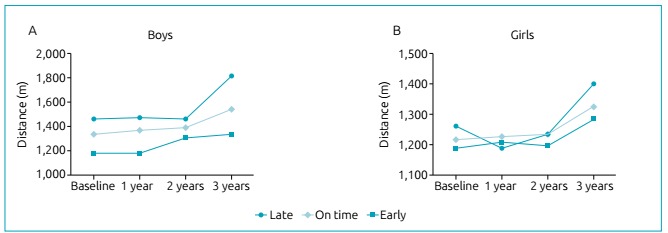
Nine-minute running distance according to follow-up and maturity status.

Tracking of cardiorespiratory fitness from childhood to adolescence was moderate to low (Figure 1). Tracking values were greater among boys [boys: k=0.357 (p<0.001), LCCC=0.521 (p<0.001); girls: k=0.224 (p<0.001), LCCC=0.338 (p<0.001)]. Moreover, the mean age of increase cardiorespiratory fitness was after 12 years (Figure 2). When analyzing the kinetics of cardiorespiratory fitness according to maturity status (Figure 3), the impact of each category is more evident among boys (Figure 3A), in which the cardiorespiratory fitness of on-time maturing adolescents increased linearly. Early maturing adolescents presented an abrupt increase at around 11 to 12 years old, and late maturing adolescents increased only in the final year of follow-up (around 12 to 13 years old). In addition, body fatness was inversely correlated with respective age at PHV and the 9-minute running test at both baseline [boys: -0.108 (0.131), -0.354 (p<0.001); girls: -0.224 (p=0.002), -0.292 (p<0.001)] and after three years of follow-up [boys: -0.305 (p<0.001), -0.539 (p<0.001); girls: -0.178 (p=0.018), -0.262 (p<0.001)].

Moreover, the interaction between cardiorespiratory fitness at baseline and age at PHV was significant in the total sample (adjusted by sex) and among boys, regardless of the effect of cohort, chronological age, and sum of skinfolds (Table 2). Differently, in girls there was no interaction between cardiorespiratory fitness and age at PHV (p=0.413).

**Table 2:** Interaction terms predicting 9-minute running at three years of follow-up.

Interactions	β	95%CI	p-value
Total sample			
9-min running at baseline × APHV	0.184	0.029 to 0.339	0.020
Boys			
9-min running at baseline × APHV	0.644	0.225 to 1.064	0.003
Girls			
9-min running at baseline × APHV	-0.217	-0.737 to 0.304	0.413

APHV: age of peak height velocity; 95%CI: 95% confidence interval. Note: models are adjusted by cohort, chronological age, sum of skinfolds at three years of follow-up and sex in the model of total sample.

## DISCUSSION

This longitudinal study analyzed the tracking of cardiorespiratory fitness at different maturational stages; our main findings were that the tracking of cardiorespiratory fitness was moderate to low among the total sample and separated by sex. Moreover, somatic maturation significantly interacted (moderated) with aerobic fitness at baseline to predict aerobic fitness in early adolescence among boys.

Cardiorespiratory fitness is a recognized protection factor associated with several negative health outcomes even during childhood and adolescence.^4^ Moreover, cardiorespiratory fitness seems to mediate the relationship between obesity and cardiometabolic risk in the transition between childhood and adolescence.^5^ Thus, to understand cardiorespiratory fitness’ tracking from childhood to adolescence can support possible effective interventions. However, during the end of childhood and beginning of adolescence, biological maturation processes occur, which affect cardiorespiratory fitness, especially among boys.^17^ In this sense, we evaluated physical fitness tracking from childhood to adolescence, as well as the interaction of biological maturation with this maintenance.

In the present study, the tracking of aerobic fitness was moderate to low from childhood to adolescence. In fact, this finding corroborates previous studies.^8^ Low tracking coefficients can be due to the sensitivity of cardiorespiratory fitness in response to many factors, such as physical exercise^18^ and obesity,^19^ as cardiorespiratory fitness tends to be underestimated among obese subjects due to the additional difficulty caused by a running test.^20^ In addition, greater rates of becoming unfit have been observed among children/adolescents with low physical activity and high screen time.^21^ However, it is important to highlight that, while almost 60% of the young people in the highest tercile at baseline remained in the highest tercile after three years, less than 12% of the young people in the lowest tercile at baseline were classified in the highest tercile after the follow-up period. In this sense, as tracking in the higher group of fitness is positive for health, tracking in the lowest tercile of cardiorespiratory fitness should be a risk factor, given the negative association between cardiorespiratory fitness and metabolic risk.^22^

Beyond the tracking of cardiorespiratory fitness, we found that biological maturation moderated the relationship between cardiorespiratory fitness in childhood and in adolescence in boys, but not in girls. Even though biological maturation was positively associated with cardiorespiratory fitness in the crude models (late maturing presented advantages), when adjusted for adiposity, biological maturation presented an inverse relationship with cardiorespiratory fitness (early maturation presented advantage). Considering that our sample was from a private school with higher income subjects, the prevalence of high adiposity levels among early maturing boys was high. Obesity can influence the cardiorespiratory fitness test itself, in which overweight and obese adolescents present greater difficulty to run,^20^ as well as the outputs, given that adiposity is not a high metabolic tissue.^23^ Despite this, we observed that cardiorespiratory fitness increased in all groups of maturity status among boys, being that early maturing boys increased earlier, while late maturing boys increased one year later, and the increase among on time maturing adolescents was constant, which is consistent with the literature.^10^ ^,^ ^17^

Our conclusions agree with previous findings that pointed out association between biological maturation and cardiorespiratory fitness among boys. Batista et al.^11^ discovered that biological maturation was associated with cardiorespiratory fitness using a sample of non-athlete boys and the association lost the significance when scaled by size descriptors, indicating that the effect of biological maturation should pass through changes in body size characteristics. Moreover, using both sexes, Armstrong et al.^24^ found that biological maturation was independent from body mass in the association with aerobic fitness. Moreover, our results corroborate the findings of Polish active adolescents, which came up that the association between puberty and cardiorespiratory fitness was significant among boys, but not girls in longitudinal analyses.^25^ In this sense, our outcomes agree with findings from countries with high socioeconomic conditions.

The increase in cardiorespiratory fitness among boys could be explained by the biological maturation process, in which there is an increase in growth hormone (GH) release and, consequently, insulin-like growth factor-1 (IGF-1) availability^26^ especially in skeletal muscle, causing hypertrophy and hyperplasia.^7^ In addition, the development of left ventricle and pulmonary evolution^27^ can improve oxygen transportation and, consequently, increase cardiorespiratory fitness.

On the other hand, even with the development of these functions during puberty among girls, the increases in relative body fatness seem to limit gains in cardiorespiratory fitness tests.^7^ These results evidence the clear sexual differentiation between boys and girls during puberty, in which increases are observed especially in the release of testosterone and estrogen, respectively.^7^ While cardiorespiratory fitness presents no differences between sexes before puberty, the impact of maturity status on physical fitness among girls is lower than in boys. Thus, boys tend to present greater cardiorespiratory fitness during and after puberty.^28^

The present study confronts some limitations that have to be pointed out. Firstly, our method of measuring cardiorespiratory fitness (9 minutes of running), which is a field test, could present bias related to the control of speed during the test by children and adolescents. The reliability is an important issue in tracking studies, because the variation among terciles along the years can be affected out of possible variations in the test conduction. However, the test presents good reliability in this population.^29^ Moreover, tests were conducted by the same staff. Our method of somatic maturity is not direct and similar methods present bias, and the equations used to find the peak height velocity present limitations with early and late maturing young people of both genders.^30^ Furthermore, we used data from one school, in which students have high socioeconomic conditions. Therefore, extrapolation of the present findings should be made with caution. On the other hand, we presented data of a 3-year follow-up study from a country in development, what we consider as a strength of the study. Finally, we did not adjust our analysis for behavioral factors such as dietary patterns and physical activity; although, we adjusted it for fatness, which is an important confounding factor.

Finally, we found that the tracking of cardiorespiratory fitness from childhood to adolescence is moderate to low in both sexes. Moreover, maturity status moderated the relationship between cardiorespiratory fitness at baseline and in adolescence, suggesting that part of tracking can be explained by biological maturation. Thus, we suggest that early interventions aiming to improve cardiorespiratory fitness should be conducted even among early ages,^18^ and late maturing adolescents should receive particular attention in order to avoid possible dropouts due to reduced physical capacities when compared to their peers of for the same chronological ages.
